# What´s in a name: the issue with regulatory definitions

**DOI:** 10.3389/fbioe.2026.1904562

**Published:** 2026-09-03

**Authors:** Clara Rubinstein

**Affiliations:** Graduate Certificate on Risk Analysis, School of Agronomy, University of Buenos Aires, Buenos Aires, Argentina

**Keywords:** genome editing, GM microorganisms, GMOs, regulatory criteria, regulatory definitions

## Abstract

The title refers to a line from William Shakespeare’s Romeo and Juliet to suggest that names are unimportant^1^. While this may hold true in the context of human equality, clear terminology and definitions are essential for legal and regulatory purposes. Clear, unambiguous definitions reduce discretionary interpretations, make regulatory requirements more predictable and transparent, and facilitate compliance. The rise of gene-editing techniques and the renewed interest in agricultural biologicals call for a revision of the criteria used to define a GMO, particularly for microorganisms.

## Introduction

Regulatory oversight systems are grounded on normative bodies that vary around the world in their formats but that are always legally binding. And the definitions used in those documents are key, since they condition the regulatory process. With the advent of precise genome modifications, it became evident that the original definition created over 25 years ago needed re-interpretation, as the resulting modifications using some of these techniques are indistinguishable from those that could be obtained through traditional breeding.

In addition to the specific GMO definition, other related terms are equally important because of their impact on regulatory outcomes. Terms such as “History of Safe Use” (HOSU), “Familiarity” and “Novel Foods” are defined differently across regulatory documents. This is significant because HOSU, for instance, has been established as a requirement for the counterparts used as comparators in regulatory studies (CODEX -CAC/GL 45-2003); it is also the basis for considering null segregants to be GMOs in Europe (see below), and it is used to waive certain requirements for expression products, provided that they have HOSU. It is common for these terms to be defined vaguely or arbitrarily and to be subject to discretionary interpretation, which underscores the need for clarification and, ideally, harmonization (Capalbo et al., 2020).

The GMO definition created 25 years ago by the Biosafety Cartagena Protocol (Secretariat of the Convention on Biological Diversity, 2000) is the base of almost all normative bodies for biotechnology around the world. It establishes the use of recombinant DNA as the main regulatory trigger (the process), also refers to modifications that could not occur in nature and is still the predominant criterion around the world, except in Canada where the product´s novelty is the trigger (Canadian Food Inspection Agency’s, 2023; Spaans et al., 2025).

Names can demonize or glorify technologies, products or foods. This has an impact not only on consumer concerns but imposes perception and political pressures on decision makers too, which in turn, may have consequences on potential innovations and their benefits to society being delayed or prevented (Hodgson, 2026). Regulatory texts should not use value laden language, but ambiguous or obscure language should be also avoided, as also the excessive use of jargon. Applicants should clearly understand what and how they should comply with. Excessively broad definitions that leave room for multiple interpretations are not fit for regulatory purposes. The following sections describe the conceptual underpinnings of these interpretations and their consequences.

### One definition, multiple interpretations

In Article 3, the Protocol defines an LMO (Living Modified Organism) as “Any living organism that possesses a novel combination of genetic material obtained through the use of modern biotechnology”^2^, being LMOs those GMOs that are capable of transferring or replicating genetic material and “modern biotechnology”, basically genetic engineering.

A “novel combination” refers to an alteration of the genetic material “that does not occur naturally through mating or natural recombination”. These definitions are the base of international regulatory guidelines, however, the criteria used for their interpretation are not uniform, which has consequences. As summarized in Table 1, interpretations can be described as “structural-molecular” when based on the use of recombinant techniques *per se* or on the presence of a construct, or “taxonomical” when more focused on the presence/expression of foreign DNA. Under the latter, cisgenics^3^ could be treated as non-GMOs even when bearing a construct.

**TABLE 1:** Different interpretations of the GMO definition can lead to different outcomes.

Regulatory trigger (derived from the definition)	Interpretation of the trigger	Outcomes/comments
Use of recombinant DNA + novel combination (process based)	Structural/molecular (construct-based) The mere use of recombinant DNA (detectable or not) defines a GMO The presence of an integrated construct expressing foreign DNA determines a GMO Taxonomical: Integration of foreign DNA (outside the species or the genus) defines a GMO/GMM	Gene editing would be GM The absence of inserts would determine conventional breeding status NGT-2 derived changes would define GMOs in the EU and other countries like Peru^4^ Cisgenics could be exempt under this criterion Some inserts (not expressing foreign DNA) could be treated as conventionally bred
Novelty (product-based)	Canada focuses on the novelty of the end product	Novel traits can be developed through various techniques, including, but not limited to, genetic engineering
Microorganisms	USEPA: Intergeneric microorganisms are considered GMM Europe: a Microorganism in which the genetic material has been altered in a way that does not occur naturally by mating and/or natural recombination	✓ The concept of species differs from plants or animals ✓ The presence of “foreign” DNA should be re-defined ✓ Natural exchange mechanisms should be considered

Defining “foreign” is another source of divergent views among agencies. Most agencies define foreign sequences if they come from outside the species (Spaans et al., 2025), however, the Environmental Protection Agency (EPA) considers genes coming from “beyond the genus” as a regulatory trigger when applied to microorganisms (Wozniak et al., 2012; Chemla et al., 2025). When regulators, at their discretion, combine structural-molecular and taxonomical criteria, even under the same definition, an identical modification may or may not be classified as a GMO.

In contrast, regulatory agencies in Canada apply a novelty criterion to trigger a regulatory process. Novelty is clearly defined in the normative: an agricultural product may be considered “novel” if it has a new trait(s) or characteristic(s), or a changed trait(s) or characteristic(s), or a new use, or have not been previously registered in the country. This concept is used across regulated products in Canada: novel foods, novel aquatic organisms and new substances (Canadian Food Inspection Agency’s, 2023).

### Definitions condition the regulatory status of precision breeding

As explained, the criteria used to define if the outcome of genetic manipulations is in fact “a living organism that possesses a combination of genetic material that does not occur naturally through mating or natural recombination” can be different.

Precision breeding techniques exemplify how discrepancies in the interpretations lead to different outcomes (See Table 1 and Discussion section). Argentina was the first country to set a criterion for these techniques back in 2015, recently updated^5^. They interpret that if these do not result in the stable integration of a construct expressing foreign DNA, they would be generally treated as conventionally bred. The use of recombinant DNA is still the trigger and needs a pre-consultation to prove that no parts of the constructs used remain. Once proved, the outcome would not be considered a GMO. This mixed approach (structural-taxonomic) has been also adopted by other agencies, excluding genome-edited plants from GMO regulations when no inserts/foreign DNA are present.

By contrast, in the European Union “once a GMO, always a GMO” has been the prevailing guiding principle, considering that null segregants (basically, the products of gene editing techniques) lack a history of safe use and consequently are not exempted from the GMO regulations. Interestingly, the recent approved guideline for New Genomic Techniques (NGTs) does consider certain types of techniques - categorized as “NGT1”- as non GMO (Regulation (EU), 2026). This allows for a limited number of modifications that do not introduce foreign DNA. However, seeds improved via NGT-1 will have to be labelled and will not be allowed in organic production, contradicting the conventionally bred status. This inconsistency will certainly have consequences on the acceptance of these products (Taylor, 2026). The NGT-2 category, on the other hand, includes modifications exceeding the limitations imposed to NGT-1 and will be subject to the GMO regulation (EC Directive 2001/18 and Regulation EC 1829/2003) which remains to be the main rule. These criteria have been challenged based on the known extensive genomic variation in the breeder´s pool and the rapid advances of the technologies (Mewett et al., 2026; Schulman et al., 2025).

Furthermore, this proposed guideline might not be uniformly adopted by Member States, which are also diverse in their interpretations of the GMO definition. De Schrijver et al. described this complexity in a recent Report (De Schrijver et al., 2024) based on inquiries to representative European stakeholders to identify areas of consensus and divergence among Member States. The report found important incongruencies in the interpretation of the Protocol definition and concludes with a call for a precise definition of GMOs, “as it delineates the scope of the GMO legislation, ensuring that all stakeholders are aware of which organisms are subject to regulation”.

### The case of microorganisms

The GMO definition in use has allowed for the development, adoption and trade of GM crops for 25 plus years. But the current interest in developing (and engineering) microorganisms for agricultural and other uses demands a profound revision, as what has worked for plants will no longer be adequate (Rubinstein et al., 2025; Karberg, 2025).

As mentioned, the EPA´s Office of Pollution Prevention and Toxics (OPPT) in the US oversees any use of “intergeneric” bacteria (Wozniak et al., 2012). This term defines an organism that contains genetic material from a different genus, that would be therefore considered a Genetically Modified Microorganism (GMM). It is unclear how regulatory sequences or genetic remnants left from constructs would lead to the designation of intergeneric (or GMM). This approach seems very limited, considering the scope of genetic exchanges among microorganisms in nature. In fact, the current intergeneric definition has been challenged, since species taxonomy is a dynamic space, possibly making inter- or intrageneric definitions outdated as new knowledge emerges, which is not predictable enough for regulatory purposes (Chemla et al. , 2025).

According to the European Directive a GMM is “a microorganism in which the genetic material has been altered in a way that does not occur naturally by mating and/or natural recombination” (European Parliament, 2009). But it is extremely difficult to define a GMM based on what “does not occur naturally”, given the genetic fluidity among microorganisms in the environment. Defining foreign DNA is equally challenging in the microbial world, making it difficult to define a GMM in regulatory terms, as “GM” microbes do exist in nature (Karberg, 2025; Rubinstein et al., 2025). This is why if a taxonomical definition for transgenic microorganisms needs to be created, it should go far beyond the genus.

Wesseler and co-authors analyzed the legal and economic aspects of the definitions used in the EU and evaluated the economic consequences of four possible regulatory models for GMM. From a EU-level regulation, to individual member states setting their own norms -potentially adopting different definitions-these models would have different consequences on innovation and trade.

The power of the definitions is evident from this analysis, as a definition can make a development accessible, it can delay it by years, or make it non-viable at all for investors (Wesseler et al., 2023). In relation to this discussion, (Lensch et al., 2024) have recently stated that “The terms GMOs and non-GMOs are no longer fit for purpose. Even more clearly than in plants, the boundaries between “genetically modified” and “conventional” microorganisms have become blurred”.

The case of microorganisms deserves a note on dual use applications (Akhila, 2025), as specific situations might raise biosecurity concerns. As discussed, the focus of the risk assessment should be put on the phenotype (traits) and intended use of a micoorganism developed for environmental release, be it engineered or not. This is why as quoted above, definitions for engineered microorganisms do not follow the same logic as for other organisms, as the boundaries are difficult to define. Going forward, the establishment of shared frameworks and collaborative systems could contribute to resolve the intersection between biosafety and biosecurity considerations of dual use innovations.

## Discussion

It can be argued that the GMO definition is not based on science, since all domesticated species have been modified to some degree by human interventions. Irrespective of the above, the definition is so broad and complex that accommodates multiple interpretations of what a GMO is, resulting in divergent outcomes and subsequent regulatory treatments (see Table 1). The “transgenic” term might be more accurate to define a novel combination based on the expression of foreign DNA -and therefore the regulatory trigger-although natural transgenics are known to exist (Fernández Ríos et al., 2025). These historical incongruencies have now surfaced and require an open, science based revision of the definitions and their interpretations, especially for microorganisms, as the current definitions are not fit for purpose.

Additionally, divergent interpretations of definitions and regulatory triggers can lead to trade impairments between countries (or even member states within the EU) that de-regulate products in asymmetric ways. When considering precision breeding and microorganisms in particular, it is important to assess the extended consequences of the lack of harmonized regulatory criteria. The growing pipeline of edited products in some countries will widen the divide with Europe and other net importers of food and commodities. Since 2019, numerous edited products have been cleared for commercialization in several countries, mostly from the Americas, like Argentina, Brazil, Chile, Canada, the US and also in Japan (mustard greens, soybean, lettuce, banana, tomato, corn, potato, strawberries and camelina). Other countries, including those which import products from the Americas, do not allow edited products or treat them as transgenic, while others are still defining their regulatory frameworks (Ricroch et al., 2026).

In the case of biologicals, this will be even more problematic for future developments, not only in terms of trade or compliance, but because it will delay the adoption of products that may be beneficial for consumers, growers, the environment and for climate change mitigation (Zimny and Sowa, 2021).

In the case of microorganisms, the process vs. product discussion becomes ever more relevant as the focus should be put on the function and mechanisms of action of the modifications and not on the methods. In this regard, the European Food Safet Agency (EFSA) has recently recommended that “the risk assessment approach of microorganisms should be based on the strain/product itself, independently of the method used to alter the genotypic or phenotypic characteristics” and confirmed this view in the new guidance on the characterization of microorganisms (EFSA et al., 2025). Whether this recommendation will translate into a more rational approach remains to be seen, as it would also challenge the historical GMO definition and its process based approach.

In sum: names do matter, as clear, fit for purpose definitions and risk assessment criteria that are not subject to discretionary interpretations, would greatly improve regulations and contribute to their transparency and predictability.

## Data Availability

The original contributions presented in the study are included in the article/supplementary material, further inquiries can be directed to the corresponding author.

## Footnotes

2
https://bch.cbd.int/protocol/text/article.shtml?a=cpb-03, Use of Terms.

3 Organisms bearing sequences or genes from the same or closely related species.

5
https://www.magyp.gob.ar/sitio/areas/biotecnologia/conabia/_pdf/Resolucion_24-2026.pdf.
